# Role of Interleukin-22 in liver diseases

**DOI:** 10.1007/s00011-014-0727-3

**Published:** 2014-03-13

**Authors:** Chun-xiao Pan, Jie Tang, Xiao-yu Wang, Fan-rong Wu, Jin-fang Ge, Fei-hu Chen

**Affiliations:** School of Pharmacy, Anhui Medical University, 81 Mei-shan Road, Hefei, 230032 China

**Keywords:** IL-22, Liver disease, Therapeutic

## Abstract

**Introduction:**

Interleukin (IL)-22, originally referred to as IL-TIF for IL-10-related T cell-derived inducible factor, is a member of the IL-10-like cytokine family. IL-22 is highly expressed by Th17 cells and is tightly linked to chronic inflammation, including inflammatory bowel disease and local intestinal inflammation among others.

**Materials and methods:**

A PubMed and Web of Science databases search was performed for studies providing evidences on the role of IL-22 in liver diseases.

**Conclusion:**

IL-22 plays an important role in ameliorating liver injury in many rodent models by targeting hepatocytes that express high levels of IL-22 receptor 1 and IL-10 receptor 2. This review concisely summarizes the role of IL-22 in the development progression of liver disease of different etiologies. It is focused mainly on the IL-22 intracellular signaling and its influence on liver diseases.

## Introduction

Common liver disease represents an important cause of mortality and morbidity. It is a rather dynamic process of wound healing in response to a variety of persistent liver injury caused by factors such as ethanol intake, chronic hepatitis B virus (HBV) and hepatitis C virus (HCV) infection, drugs, toxins, cholestasis, and metabolic disorders [1]. Liver fibrogenesis is a common widespread pathological damage and the initial results can be amplified by an inflammatory response. Inflammatory responses upon liver injury comprise resident as well as infiltrating immune cells [2]. It is well known that innate immune cells are important triggers of hepatic inflammation because the liver is selectively enriched with macrophages (Kupffer cells), natural killer (NK), and natural killer T (NKT) cells [3].

Interleukin (IL)-22 is an IL-10 family cytokine member produced primarily by T-helper (Th)17, Th22, and NK cells [4, 5] (Table 1). IL-22 is highly expressed by IL-17-producing cells, called Th17 cells [6, 7]. In addition to Th17 cells, Th22 cells, a subset of CD4 + T cells that specifically express IL-22 and are mainly found in tissues, produce IL-22 in response to IL-6 and tumor necrosis factor-α (TNF-α), particularly in the skin [8]. IL-22 is also produced by oligoclonal γδT cells that are detected in the skin epidermis, gut, and lung epithelia [9]. NK cells produce IL-22 in response to IL-12 and IL-18 or IL-23 [10]. In addition, retinoic acid-related orphan receptor gtpositive (RORγt+) innate lymphoid cells, including lymphoid tissue inducer (LTi) and LTi-like cells, also express IL-22. LTi-like cells express low constitutive levels of IL-22 that are highly induced upon activation by IL-23 stimulation [11].

**Table 1:** The main cell source of IL-22

Cell type	Location	Transcription factor	Stimulating cytokine	References
Th1 (human)	Blood	Aryl hydrocarbon receptor (AHR)	IL-12	[5]
Th17	Skin, intestine, lung	Retoinic acid-related orphan receptor (ROR) γt (mouse), RORC (human)	IL-6	[6]
Th22	Skin	AHR	IL-6, TNF-α	[8]
γδT cells	Skin, intestine, lung	RORγt (mouse)	IL-1β1, IL-23	[9]
Natural killer T (NKT) cells	Skin, intestine, lung	Retoinic acid-related orphan receptor (ROR)	IL-12, IL-18, IL-23	[10]
Lymphoid tissue inducer (LTi) cells	Spleen	Retinoic acid-related orphan nuclear hormone receptor C (RORC)	IL-23	[11]

IL-22 has been shown to be an important mediator in dermal inflammation, and has protected mice from inflammatory bowel disease (IBD) [12]. In patients with IBD, diseases of the liver and the biliary tract are commonly observed. Moreover, drug-induced hepatotoxicity and non-alcoholic fatty liver disease (NAFLD) are the most frequent liver complications in IBD [13]. Therefore, IL-22 can provide protection to hepatocytes during liver inflammation. In this review, we summarize our current understanding of the involvement of IL-22 in different development and pathogenesis pathways of liver diseases, as well as its clinical implications and therapeutic potential.

## IL-22 and IL-22R and their biological functions

IL-22 is a member of the IL-10 cytokine family, initially described in 2000 by Dumoutier et al. [14], and originally referred to as IL-10-related T cell-derived inducible factor (IL-TIF). The IL-10 family members share only a limited primary sequence identity of about 13–25 %, but display a common gene structure, a similar secondary protein structure, and the use of the same receptor family. IL-22 shares 22 % amino acid sequence identity with IL-10 [15]. Human IL-22 protein is 146 amino acids in length and has 80.8 % identity with murine IL-22. Like other IL-10 family members, the IL-22 structure contains six α-helices (referred to as helices A to F) [16].

IL-22 exerts its biological activities through the interaction with a heterodimeric receptor complex composed of IL-10 receptor 2 and the more restricted IL-22 receptor 1, which is mainly expressed on intestinal and respiratory epithelial cells, keratinocytes, and hepatocytes, but not on cells of hematopoietic origin [17]. IL-22 binding to the IL-22R complex induces a cascade of downstream signaling pathways. The initial studies using a murine kidney cell line demonstrated that IL-22R ligation activated the transcription 3 (STAT3) pathway, and to a lesser extent, STAT5 phosphorylation, while other studies reported that IL-22R ligation activated the phosphorylation of STAT1, STAT3, and STAT5 in a human kidney cell line. Using the H4IIE rat hepatoma cell line, Lejeune et al. [18] investigated that IL-22 signaling utilized c-Jun N-terminal kinase (Jak1) and tyrosine kinase (Tyk2) to propagate downstream phosphorylation signals, including mitogen-activated protein kinase (MAPK) signaling pathways [p38 kinase, c-Jun N-terminal kinase (JNK), and extra cellular-signal-regulated kinase 1/2 (ERK1/2), STAT1, STAT3, and STAT5].

STAT3 signaling leads to the induction of genes involved in many processes, including apoptosis, cell-cycle progression, and angiogenesis. STAT3 is critical to proper mouse development, and STAT3-deficient mice died early in embryogenesis. Although liver-specific STAT3-deficient mice had no abnormalities in liver development, they did have a reduced ability to recover from liver damage [19]. Thus, activation of STAT3 and subsequent induction of a variety of anti-apoptotic and proliferation-associated genes seemed to contribute to the hepatoprotective and mitogenic effect of IL-22 in the liver. The elevated IL-22 stimulated STAT3 activation in hepatocytes and subsequently upregulated the expression of anti-apoptotic genes (e.g., B-cell lymphoma-2 family, Bcl-2), anti-oxidative genes (e.g., metallothioneins 1 and 2), and mitochondrial DNA repaired genes (e.g., 8-oxoguanine DNA glycosylase 1, OGG1 and Nei-like homolog 1, Neil 1), and downregulated the expression of lipogenic genes (e.g., sterol regulatory element-binding proteins, SREBP-1c) [20]. Moreover, blocking or deletion of STAT3 activation abolished the antiapoptotic and mitogenic actions of IL-22 in hepatic cells, whereas the overexpression of a constitutively activated form of STAT3 promoted hepatic stellate cell (HSCs) senescence via p53- and p21-dependent pathways [21].

In view of the biological effects, IL-22 seems to be a novel type of immune mediator. It does not affect immune cells but regulates functions of certain tissue cells, although it is produced by immune cells [17]. In summary, the functions of IL-22 are (1) sustaining the integrity and barrier functions of the tissues and (2) preventing damage caused by either invading pathogens or the inflammatory response itself [5, 22, 23]. In this process, IL-22 can directly increase the innate immunity of tissue cells, protect tissues from damage, and enhance their regeneration. IL-22 is a well-documented antioxidant factor for hepatocytes via the upregulation of anti-oxidative genes (e.g., metallothioneins 1 and 2) [24]. Finally, IL-22 treatment is a potential adjunct therapy strategy for treating severe forms of diseases because of its antiapoptotic, anti-steatotic, antifungal [25], and antimicrobial effects, as well as the potential added benefit of few side effects [24].

## IL-22 in the pathogenesis of liver disease

### T cell-mediated hepatitis

Concanavalin A (Con A)-induced liver injury is a well-established model of T-cell-mediated hepatitis [26]. Within 8–24 h, the administration of this mitogenic lectin resulted in rapid liver inflammation and necrosis, and many features of Con A injury were believed to resemble human autoimmune liver disorders [27, 28]. Liver injury from large doses of Con A was the result of a number of immune cell-mediated and cytokine-driven responses and was dependent upon activation of CD4 + T cells, as the depletion of these cells ameliorates hepatic injury [29]. It is reported that after injection of Con A, IL-22 protein expression was significantly increased, peaking between 1 and 9 h and returning to basal levels at 24 h. IL-22 mRNA expression was significantly increased by Con A injection in the intact liver, hepatic mononuclear cells, and purified CD3 + T cells [30]. IL-22 blockade by neutralizing antibody or genetic deletion markedly exacerbated liver injury in this model [10], while the administration of recombinant IL-22 prevented Con A-induced liver injury. Injection of IL-22 rapidly induced STAT3 activation in the liver, and IL-22 blockade significantly reduced hepatic STAT3 activation in T-cell-mediated hepatitis, indicating that IL-22 is also partially responsible for hepatic STAT3 activation in this model [20]. Activation of STAT3 subsequently led to the upregulation of a variety of anti-apoptotic (e.g., Bcl-2, Bcl-xL, Mcl-1) and mitogenic (e.g., c-myc, cyclin D1, Rb2, CDK4) genes, which resulted in hepatoprotective effects under conditions of liver injury. Moreover, IL-22-transgenic mice with an overexpression of IL-22 were completely resistant to Con A-induced liver injury, while IL-22-deficient mice were highly susceptible to such injury.

### Toxic liver injury

Carbon tetrachloride (CCl4)-induced liver fibrosis is a well-established model in terms of its detrimental effects for drug-induced liver injury and can be used in the clinic to examine anti-hepatotoxic and/or hepatoprotective drugs [1]. The Bin Gao groups revealed that overexpression of IL-22 by either gene targeting (e.g., IL-22 transgenic mice) or exogenous administration of adenovirus-expressing-IL-22 (Ad-IL-22) could reduce liver fibrosis and accelerate the resolution of CCl4-induced liver fibrosis during recovery. Furthermore, IL-22 overexpression or treatment of IL-22 could increase the number of senescence-associated beta-galactosidase-positive HSCs and decrease the expression of alpha-smooth muscle actin in fibrotic livers in vivo and in vitro. In vitro treatment with IL-22 induced the activation of STAT3 in primary mouse and human HSCs. Deletion of STAT3 prevented IL-22-induced HSCs senescence in vitro, whereas the overexpression of a constitutively activated form of STAT3 promoted HSC senescence through p53- and p21-dependent pathways. Finally, IL-22 treatment upregulated the suppressor of cytokine signaling (SOCS) 3 expression in HSCs. Immunoprecipitation analyses revealed that SOCS3 bound to p53 and subsequently increased the expression of p53 and its target genes, contributing to IL-22-mediated HSCs senescence [31]. However, the study also declared that the IL-22-mediated inhibition of liver fibrosis could not be completely mediated via its hepatoprotective effects because IL-22 liver-specific transgenic (IL-22TG) mice displayed a similar extent of liver injury after chronic CCl4-treatment when compared with wild-type (WT) mice [31].

### Acute liver injury induced by acetaminophen or thioacetamide

Besides viral infections, acetaminophen (APAP, paracetamol)-induced hepatotoxicity is a major cause of hepatotoxicity and acute liver injury worldwide. Studies showed that the therapeutic efficacy of recombinant IL-22 was assessed in the context of APAP-induced hepatotoxicity. IL-22 application was associated with the amelioration of histopathologic damage, reduction of serum alanine aminotransferase activity, hepatic induction of prototypic STAT3-inducible genes, and a tendency, albeit not reaching significance in the set of experiments performed, toward reduced mortality in otherwise fatal intoxication. Because systemically applied IL-22 might not be associated with acute immunomodulation/stimulation, therapeutic activation of the IL-22-STAT3 axis may provide surplus therapeutic benefit for hard-to-treat patients, for whom N-acetylcysteine frequently falls short.

In the thioacetamide-treated liver, IL-10 gene therapy reversed hepatic fibrosis and prevented cell apoptosis. Following gene transfer, the activation of α-smooth muscle actin and cyclooxygenase-2 was significantly attenuated [32]. However, the role of IL-22 in this model is unclear.

### Alcoholic liver disease

Chronic alcohol drinking is a major cause of chronic liver disease worldwide which results in a spectrum of liver disorders that range from simple fatty liver to more severe forms of liver injury such as alcoholic hepatitis, cirrhosis, and hepatocellular carcinoma [33, 34]. Many mechanisms underlying the pathogenesis of ALD including direct hepatotoxicity of ethanol and its metabolites, oxidative stress generated by ethanol metabolism, activation of innate immunity, elevation of pro-inflammatory cytokines and chemokines have been identified [35–38]. Regarding the infiltration of leukocytes into inflamed liver tissue, T cells have been described as a major part of the inflammatory response following alcohol-induced liver injury, showing high activity, for example by secreting a variety of inflammatory cytokines such as IL-1β, IL-6, and TNF-α in vitro after isolation [39–43]. Current therapeutic options for alcoholic hepatitis include corticosteroids or TNF-α inhibitor therapy; however, these treatments have generated controversial results and are associated with increased rates of infection.

IL-22 treatment ameliorated steatosis and liver damage in several models of liver injury, including chronic-binge ethanol feeding, acute ethanol feeding, and high-fat diet-induced fatty liver disease. Bataller R et al. [21] verified that the hepatoprotective effect of IL-22 in alcoholic liver injury was mediated the upregulation of anti-apoptotic genes, anti-oxidative genes and the downregulation of lipogenic genes in hepatocytes via the activation of STAT3 signaling pathway. Overexpression of IL-22 in vivo or in vitro promoted liver regeneration or hepatocyte proliferation, respectively. Additionally, IL-22 treatment may potentially augment liver repair by promoting LPC proliferation and survival. Therefore, co-treatment with IL-22 may diminish the side effect and prevent the inhibition of liver regeneration because IL-22 has an antimicrobial effect [44] and can promote liver regeneration [30, 45]. Furthermore, hepatic expression of IL-22R1 was upregulated in patients with alcoholic hepatitis without elevation of IL-22, suggesting that these patients may be sensitive to IL-22 treatment.

### Nonalcoholic fatty liver disease

Nonalcoholic fatty liver disease is one of the most common liver diseases in the Western world, with an estimated prevalence of 17–33 %. It is reported that treatment with oleic acid alone can induce a 4.8-fold increase of IL-22 mRNA compared to non-steatotic controls, and stimulation with glycochenodeoxycholic acid (20 μmol/L) for 1 h can induce a 10.7-fold increase of IL-22 mRNA compared to unstimulated controls [46]. Studies showed that IL-22 administration reduced high-fat-diet (HFD)-induced elevation of serum alanine aminotransferase and aspartate aminotransferase (AST) levels, and partially inhibited HFD-induced upregulation of lipogenesis-related genes that are involved in lipid synthesis in the liver. Additionally, IL-22 treatment prevented liver injury in mouse models via the activation of STAT3, whose role in protecting against liver injury has been well documented. Moreover, Yang et al. [47] recently reported that IL-22 treatment can ameliorate obesity-associated fatty liver by downregulating several lipogenesis- and triglyceride synthesis-related genes. Moreover, the activation of STAT3 by recombinant murine IL-22 (rmIL-22) was reduced by the overexpression of a dominant negative IL-22R1 in this model. The levels of triglyceride and cholesterol in the liver were significantly reduced by long-term treatment of rmIL-22 in C57 black 6 (C57BL/6) and obese (ob/ob) mice fed with HFD. The HFD-induced increases of ALT and AST in ob/ob mice were ameliorated by rmIL-22 administration. In addition, the expression of fatty acid synthase and TNF-α in the liver was decreased by long-term rmIL-22 administration.

### HBV hepatitis

Chronic hepatitis B is a complex heterogeneous disease that progresses through different phases of immune tolerance (high HBV and low ALT), immune activity (high HBV and high ALT), and immune inactivity (low HBV and low ALT) [42]. Unlike chemically-induced liver injury, the liver inflammation in the HBV-transgenic mouse T-cell adoptive transfer model is potentiated by the recruitment of inflammatory cells into the liver. This recruitment requires specific cellular and protein mediators, including neutrophils, chemokines, and matrix metalloproteinases, all of which can be induced by IL-22 [48].

Current studies demonstrated that CD3^+^T activated NKT and NK cells, which were the major cell types that produce IL-22 in the HBV-infected livers [49]. IL-22 may play a proinflammatory role during acute HBV infection, perhaps by amplifying immune cell infiltration and clearance of virus, whereas it may play a more protective role during chronic HBV infection [50]. In a model of HBV replication in HBV transgenic mice, blocking IL-22 with a neutralizing antibody ameliorated liver damage by reducing chemokine expression on hepatocytes, and subsequently preventing hepatic recruitment of inflammatory cells, suggesting that IL-22 may contribute to the pathogenesis of HBV-mediated liver inflammation and injury. In chronic Hepatitis B (HBV) infection, recent reports indicated that IL-22 was an important factor that regulates genes responsible for cell proliferation, survival, and tissue repair, consistent with its protective roles in certain contexts. Chronic HBV infection was associated with the accumulation of IL-22-producing inflammatory cells in the liver.

Although IL-22 has been reported to have no antiviral activity against HBV replication, it appears to be a mediator of the inflammatory response after recognition of HBV by T cells in the liver and is a well-documented survival factor for hepatocytes via the upregulation of anti-apoptotic and antioxidant genes. Thus, in addition to the ability of IL-22 to promote hepatocyte survival and proliferation, the accumulation of IL-22-producing inflammatory cells may also play a critical role in liver stem cell (LPCs) proliferation via the activation of STAT3 in chronic HBV infection [51]. IL-22 also increases the proinflammatory activity of TNF-α, which is expressed in the liver after transfer of HBV-specific T cells. Taken together, IL-22 may directly or indirectly contribute to liver disease pathogenesis by promoting the migration of inflammatory cells into the liver, which can increase T cell-induced hepatocyte injury.

### HCV Hepatitis

Infection with HCV is the major cause of chronic liver disease, with an estimated global prevalence of 2.5 %, which means 170 million people infected worldwide. The serum levels of IL-22 in HCV were similar to the control group. IL-22 mRNA expression is significantly higher in HCV hepatitis than in cholestatic liver disease. However, IL-22 did not directly regulate antiviral proteins and had no effect on HCV replication, because IL-22 did not influence the antiviral protein expression of MxA and 2’, 5’-oligoadenylates synthesis [52].

Dengue is a disease whose characteristics symptoms include fever, back pain, retro-orbital pain, and rash, as well as signs of liver involvement, usually detected by slightly elevated aminotransferase levels [53]. DENV-2 infection led to a significant increase in the serum levels of neutrophils, chemokine (C-X-C motif) ligand1 (CXCL1/KC), interferon and chemokine (C–C motif) ligand5 (CCL5/RANTES) in the liver of infected mice. NK cells (CD3-NK1.1 +) were the main source of IL-22 in the liver upon DENV-2 infection at day 6. Furthermore, liver sections of infected WT mice revealed signs of congestion, haemorrhage, hepatocyte degeneration, and necrosis at day 6 p.i. However, there was more serious liver damage with the deletion of IL-22 (IL-22^−/−^) upon DENV-2 infection, with increased levels of transaminases in serum, greater liver myeloperoxidase activity and exacerbated pathological damage. The production of cytokine and chemokine was also higher in the liver of infected IL-22^−/−^ mice [54].

### Hepatocellular carcinoma

Hepatocellular carcinoma is the fourth most common malignancy worldwide with around 700,000 new cases each year. In Edmondson Grade III–IV HCC patients, serum IL-22 is significantly upregulated compared with controls. IL-22 is excessively expressed in human HCC tumor-infiltrated leukocytes (TILs) compared to peripheral lymphocytes. IL-22 receptors are highly expressed in both liver cancer and adjacent cirrhotic tissues compared to normal controls. Enhanced tumor growth and metastasis were found in mice that underwent subrenal transplantation of MHCC-97H cells (hepatocellular cancer cells) co-transplanted with IL-221 TILs. Jiang et al. [55] indicated that in-vitro studies confirmed the effect of IL-22 on the tumor-promoting and antiapoptotic cells, which is similar to IL-6. In the HCC model, sustained and increased IL-22 expression and STAT3 activation were found in liver tissues. Taken together, professors suggested that serum IL-22 levels could be a negative prognostic indicator in patients with HBV-related HCC, and that IL-22 should not be used in patients with pre-cancerous cirrhosis or liver cancer.

### Liver ischemia-reperfusion injury and liver transplantation

Liver ischemia–reperfusion injury (IRI) is a major complication of hemorrhagic shock, liver resection, and transplantation. A study presented by Paul et al. described that significantly increased mRNA levels coding for IL-22 were detected at 24 h, and that the expression of IL-22R1 was increased by 6 h of reperfusion in mice that were protected from IRI. Treatment of WT mice with recombinant IL-22 markedly ameliorated serum aspartate aminotransferase levels, moderated cardinal histological features of IR damage (Suzuki’s score), and diminished leukocyte sequestration, along with the expression of IL-22R1 and pro-inflammatory cytokines. IL-22 antibody does not appreciably affect IRI but increases IL-22R1 transcription in the liver. Administration of IL-22 protein exerts hepatoprotection by STAT3 activation. Treatment with IL-22 protein may represent a novel therapeutic strategy to prevent liver IRI in transplant recipients [56].

In a liver regeneration model, the levels of serum IL-22 protein and hepatic IL-22R1 mRNA expression are significantly increased after 70 % partial hepatectomy. Blockage of IL-22 with administration of an anti-IL-22 antibody before partial hepatectomy significantly decreased hepatocytes proliferation [45].

## Therapeutic implications of IL-22 in liver disease

Presently, increasing evidence in animal models and humans have demonstrated the importance of IL-22 in the initiation and maintenance of liver disease. IL-22 may have promise as a potential therapeutic agent for liver diseases. With the continued understanding of the molecular mechanisms of IL-22, together with knowledge on the capacity of current drugs to target this pathological process, further research may open an avenue to novel therapeutic options.

**Fig. 1:**
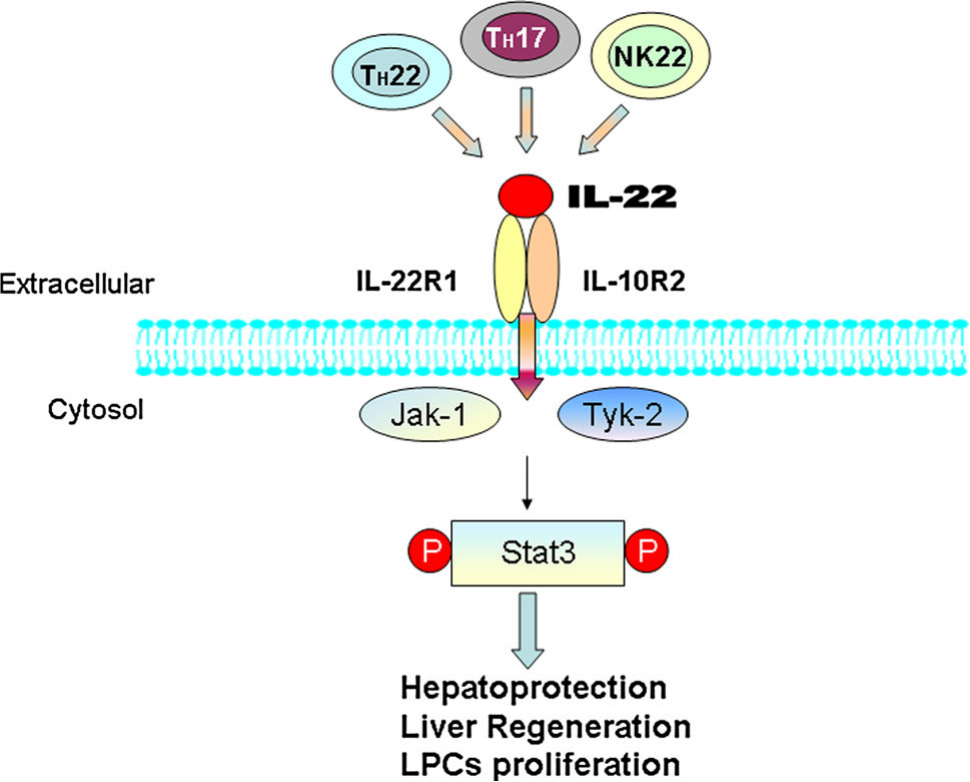
Cellular sources and effects of IL-22. Activated Th cell and NK cell subsets secrete IL-22, this cytokine acts exclusively on certain tissue cells, especially epithelial cells. IL-22 affects these target cells via the IL-22 heterodimeric receptor complex consisting of IL-22R1 and IL-10R2. These interactions lead to signal transduction via JAK–STAT pathways that mainly include the activation of Jak1, Tyk2, and STAT3. In hepatocytes, IL-22 induces STAT3 activation and plays important roles in hepatoprotection and liver regeneration

IL-22 administration ameliorated liver damage in almost all models of liver injury. Overexpression of IL-22 in vivo or in vitro promoted liver regeneration or hepatocyte proliferation, respectively. IL-22 played dual roles in controlling liver inflammation: inhibiting liver inflammation by preventing hepatocyte damage, and subsequently reducing necrosis-associated liver inflammation. Besides, IL-22 treatment may potentially augment liver repair by promoting LPC proliferation and survival in a STAT3-dependent manner (Fig. 1). Finally, more importantly, IL-22 therapy may have fewer side effects than conventional therapy, due to the restricted expression of IL-22R1 on epithelial cells (e.g., hepatocytes) and HSCs. All these results indicated that IL-22 may be an more effective and safer therapy for viral hepatitis and other liver diseases. However, several points should be emphasized. First, although IL-22 itself does not initiate liver tumor development, IL-22 is able to promote existing liver cancer cell proliferation and survival. Thus, it should not be used in patients with pre-cancerous cirrhosis or liver cancer. Second, IL-22 may play a proinflammatory role during acute HBV infection, perhaps by amplifying immune cell infiltration and clearance of the virus, whereas it may play a more protective role during chronic HBV infection.

## Conclusions

Cytokines are attractive therapeutic targets in inflammatory diseases because of their extracellular location and their importance in regulating immune and inflammatory responses. IL-22 has recently been considered to be another important hepatoprotective cytokine because of its well-documented hepatoprotective and antimicrobial functions, as well as its potentially minimal adverse side effects. Therefore, clinical trials examining combination therapy with IL-22 for the treatment of patients with liver disease are warranted.
